# MXene@c-MWCNT Adhesive Silica Nanofiber Membranes Enhancing Electromagnetic Interference Shielding and Thermal Insulation Performance in Extreme Environments

**DOI:** 10.1007/s40820-024-01398-1

**Published:** 2024-05-14

**Authors:** Ziyuan Han, Yutao Niu, Xuetao Shi, Duo Pan, Hu Liu, Hua Qiu, Weihua Chen, Ben Bin Xu, Zeinhom M. El-Bahy, Hua Hou, Eman Ramadan Elsharkawy, Mohammed A. Amin, Chuntai Liu, Zhanhu Guo

**Affiliations:** 1 Key Laboratory of Materials Processing and Mold (Zhengzhou University), Ministry of Education, National Engineering Research Center for Advanced Polymer Processing Technology, Zhengzhou University, Zhengzhou, 450002 People’s Republic of China; 2 School of Nano-Tech and Nano-Bionics, University of Science and Technology of China, Hefei, 230026 People’s Republic of China; 3 Key Laboratory of Multifunctional Nanomaterials and Smart Systems, Advanced Materials Division, Suzhou Institute of Nano-Tech and Nano-Bionics, Chinese Academy of Sciences, Suzhou, 215123 People’s Republic of China; 4 Shaanxi Key Laboratory of Macromolecular Science and Technology, School of Chemistry and Chemical Engineering, Northwestern Polytechnical University, Xi’an, 710072 People’s Republic of China; 5 College of Chemistry & Green Catalysis Center, Zhengzhou University, Zhengzhou, 450001 People’s Republic of China; 6 Mechanical and Construction Engineering, Faculty of Engineering and Environment, Northumbria University, Newcastle Upon Tyne, NE1 8 UK; 7 Department of Chemistry, Faculty of Science, Al-Azhar University, Nasr City, 11884 Cairo Egypt; 8 Department of Chemistry, Faculty of Science, Northern Border University, Arar, Saudi Arabia; 9 Department of Chemistry, College of Science, Taif University, P.O. Box 11099, 21944 Taif, Saudi Arabia

**Keywords:** SiO_2_ nanofiber membranes, MXene@c-MWCNT, Composite film, Thermal insulation, Electromagnetic interference shielding

## Abstract

A lightweight flexible thermally stable composite is fabricated by combining silica nanofiber membranes (SNM) with MXene@c-MWCNT hybrid film. The flexible SNM with outstanding thermal insulation are prepared from tetraethyl orthosilicate hydrolysis and condensation by electrospinning and high-temperature calcination; the MXene@c-MWCNT_x:y_ films are prepared by vacuum filtration technology. In particular, the SNM and MXene@c-MWCNT_6:4_ as one unit layer (SMC_1_) are bonded together with 5 wt% polyvinyl alcohol (PVA) solution, which exhibits low thermal conductivity (0.066 W m^−1^ K^−1^) and good electromagnetic interference (EMI) shielding performance (average EMI SE_T_, 37.8 dB). With the increase in functional unit layer, the overall thermal insulation performance of the whole composite film (SMC_x_) remains stable, and EMI shielding performance is greatly improved, especially for SMC_3_ with three unit layers, the average EMI SE_T_ is as high as 55.4 dB. In addition, the organic combination of rigid SNM and tough MXene@c-MWCNT_6:4_ makes SMC_x_ exhibit good mechanical tensile strength. Importantly, SMC_x_ exhibit stable EMI shielding and excellent thermal insulation even in extreme heat and cold environment. Therefore, this work provides a novel design idea and important reference value for EMI shielding and thermal insulation components used in extreme environmental protection equipment in the future.

**Supplementary Information:**

The online version contains supplementary material available at 10.1007/s40820-024-01398-1.

## Highlights

- The SiO_2_ nanofiber membranes and MXene@c-MWCNT_6:4_ as one unit layer (SMC_1_) were bonded together with 5 wt% PVA solution.
- When the structural unit is increased to three layers, the resulting SMC_3_ has an average electromagnetic interference SE_T_ of 55.4 dB and a low thermal conductivity of 0.062 W m^−1^ K^−1^.
- SMC_x_ exhibit stable electromagnetic interference shielding and excellent thermal insulation even in extreme heat and cold environment.

**Supplementary Information:**

The online version contains supplementary material available at 10.1007/s40820-024-01398-1.

## Graphical abstract

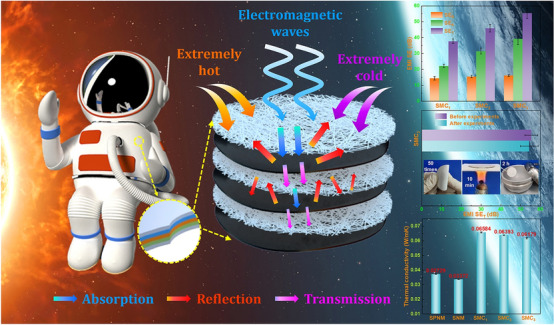

## Introduction

In recent years, manned spaceflight has become an important symbol to measure a country's scientific and technological strength. In the face of the extreme environment of space with large temperature difference, strong radiation and high vacuum, the extra-vehicular space suit has become the necessary protective equipment for astronauts to go outside the space station and carry out various kinds of work [1, 2]. For a complete space suit, the previous thermal control system and radiation protection system are relatively independent, and the design materials are complex and heavy, which seriously restrict the physical mobility of astronauts. Therefore, the development of lightweight, flexible, low-cost materials with both electromagnetic interference (EMI) shielding and thermal insulation is the key to ensure the normal life and work of astronauts in space [3, 4].

Conventional thermal insulation materials are mainly divided into foam-based materials [5], phase change materials [6] and ceramic aerogel materials [7]. Among them, foam-based thermal insulation materials have defects such as low ignition point and release of toxic substances during combustion. For most phase change insulation materials, maintaining long-lasting insulation performance requires a large space volume due to fixed enthalpy values. Ceramic aerogel materials have low thermal conductivity and slow phonon transfer rate; especially for silica (SiO_2_) aerogel, it has small pore size, high porosity and thermal stability, so it is a lightweight and efficient thermal insulation material [8, 9]. However, in practical applications, researchers found that SiO_2_ aerogel is highly transparent to infrared radiation and has great brittleness [10]. With the development of nanotechnology in recent years, effective progress has been made in converting SiO_2_ sol into flexible SiO_2_ nanofiber membranes (SNM), and the obtained SNM shows excellent heat insulation and good thermal stability based on its special pore structure [11, 12]. Si et al. [13] successfully synthesized ultra-softness SNM, which not only have excellent tensile strength of 5.5 MPa, but also exhibit ultra-low thermal conductivity of 0.0058 W m^−1^ K^−1^. Currently, the ways to obtain SNM include laser ablation [14], sol–gel [15], vapor deposition [16] and electrospinning [17]. Compared with other methods, electrospinning has the advantages of simple operation, low cost and good controllability [18–20].

In terms of EMI shielding, MXene, a two-dimensional structural material with high electrical conductivity, has been widely studied [21–23]. However, poor mechanical, chemical and thermal stability greatly limits its application range [24]. Carbon nanotube (CNT) has high aspect ratio, low density, outstanding mechanical properties, high electrical conductivity, and good chemical stability [25–27]; therefore, it is another ideal EMI shielding conductive filler; unfortunately, weak dispersion has always been a problem [28]. It has been found that the combination of MXene and CNT by special means can not only overcome the defects of each other, but also make the hybrid fillers have good comprehensive properties [29]. For example, Zhou et al. [30] combined MXene and CNT uniformly through vacuum-assisted filtration and demonstrated good EMI shielding performance and high tensile strength and toughness in the obtained MXene/CNT films. In fact, conductive fillers such as MXene and CNT have considerable thermal conductivity [31–33], so how to combine them with thermal insulation materials and coordinate EMI shielding and thermal insulation performance is always a challenge in the design of aerospace protective suits.

In this work, SiO_2_ nanofiber membranes (SNM), which mainly play an EMI shielding function, were successfully prepared by electrospinning of tetraethyl orthosilicate hydrolyzed precursor followed with a high-temperature calcination condensation process. As a component of EMI shielding function, MXene@c-MWCNT_x:y_ is obtained through vacuum filtration with MXene/c-MWCNT of different hybrid ratios, and it is found that when the mass ratio of MXene to c-MWCNT is 6:4, MXene@c-MWCNT_6:4_ has the optimal mechanical and functional properties. Then, SNM and MXene@c-MWCNT_6:4_ are effectively formed into an organic whole by cleverly using 5 wt% polyvinyl alcohol (PVA) as a binder, and this structural unit (SNM/MXene@c-MWCNT_6:4_, SMC_1_) exhibits excellent EMI shielding and heat insulation properties. When the structural unit is increased to three layers, the resulting SMC_3_ has an average EMI SE_T_ of 55.4 dB and a low thermal conductivity of 0.062 W m^−1^ K^−1^. More importantly, the resulting functional composite film (SMC_x_) resolutely has a stable EMI shielding and thermal insulation properties in simulated high-temperature and cold extreme environments. In conclusion, the design of this study not only effectively avoids the influence of MXene@c-MWCNT on the overall thermal insulation performance of the composite film by adjusting the number of functional unit layers, but also greatly improves the overall mechanical and EMI shielding performance; therefore, the composite functional film obtained in this work has broad application prospects in extreme fields like aerospace.

## Materials and Methods

### Materials

Poly(vinyl alcohol) (PVA 1788), lithium fluoride (LiF, ≥ 99.9%), hydrochloric acid (HCl, 35%) and sodium dodecyl sulfate (SDS, AR) were obtained from Shanghai Macklin Biochemical Co., Ltd. Tetraethyl orthosilicate (TEOS, 98%) was purchased from Tianjin Kemiou Chemical Reagent Co., Ltd. Oxalic acid (H_2_C_2_O_4_, AR) was provided by Tianjin Damao Chemical Reagent Factory. Ti_3_AlC_2_ (MAX) powder (≤ 38 μm, 98%) was supplied by 11 Technology Co., Ltd. Carboxylated multi-wall carbon nanotubes (c-MWCNT, ≥ 98%) were provided by Shenzhen Suiheng Technology Co., Ltd. Deionized water was supplied in unlimited quantities by the laboratory.

### Preparation of SNM

Figure S1(I) shows the preparation process of SiO_2_ nanofiber membranes (SNM). First, TEOS, H_2_O and H_2_C_2_O_4_ were mixed and stirred at room temperature at a molar ratio of 1:8.063:0.0186 for 10 h to prepare SiO_2_ precursor sol. Second, SiO_2_ sol and 10 wt% PVA solution were mixed at a mass ratio of 1:1 and stirred for 10 h to obtain the spinnable precursor solution [34]. Then, the SiO_2_/PVA nanofiber membranes (SPNM) were fabricated by an electrospinning device and the corresponding setting parameters were as follows: applied voltage of 14 kV, syringe boost speed of 1.1 mL h^−1^, drum rotation speed of 140 rpm, receiving distance of 20 cm, and the relative humidity and temperature were 40%-50% and 23–25 °C, respectively [35]. Finally, SPNM were pretreated in a vacuum oven at 60 °C and then placed in a tube furnace (BTF-1700C-CVD from Anhui BEQ Equipment Technology Co., Ltd.) with a constant heating rate of 5 °C min^−1^ up to 800 °C for 2 h to form SNM.

### Preparation of MXene@c-MWCNT_x:y_

MXene flakes were obtained by etching Ti_3_AlC_2_ MAX powder with HF. Briefly, 2 g LiF was dissolved in 100 mL HCl solution (12 M) being stirred at 35 °C for 10 min. Then, Ti_3_AlC_2_ powders were gradually added to the above mixture, and the reaction was held at 35 °C for 24 h. After reaction, the solid was separated with supernatant via centrifugation for 10 min at 3500 rpm. Subsequently, it was washed by deionized water and centrifuged until the pH = 6 [30]. The resulting Ti_3_C_2_T_x_ MXene was then dispersed in deionized water to obtain 1 mg mL^−1^ MXene dispersion.

In order to improve the solution dispersibility of c-MWCNT, c-MWCNT and a little SDS were added to deionized water and sonicated for 30 min to prepare 1 mg mL^−1^ c-MWCNT dispersion [36]. As shown in Fig. S1(II), the above two dispersions were mixed in mass ratios of 0:10, 4:6, 5:5, 6:4, and 10:0, respectively, followed by ultrasound for 30 min to prepare mixed dispersion. In the end, 50 mL mixed dispersion was filtered to prepare MXene@c-MWCNT_x:y_ with a diameter of 4 cm and a mass of 50 mg by vacuum filtration method.

### Preparation of SMC_x_

Figure S1(III) presents the preparation process of SMC_x_. Wisely, 5 wt% PVA solution was evenly spread on the MXene@c-MWCNT_x:y_, followed by that SNM was laid flat on it and tightly bonded the two layers under a certain pressure. After that, the obtained composite film was placed in the oven at 60 °C for 2 h to obtain SMC_1_. Finally, taking SMC_1_ as a unit structure, SMC_2_ and SMC_3_ with two and three SMC_1_ were obtained by the same bonding method.

### Characterization

The morphology and microstructure were observed by SEM (FESEM thermoscientific Apreo C, America). Attenuated total reflection Fourier transform infrared (ATR-FTIR) spectra in the frequency region of 4000–400 cm^−1^ at a 4 cm^−1^ resolution were recorded using an FTIR spectrometer (Nicolet 6700, America) with 32 scans. The thermogravimetric analysis (TGA) of the precursor nanofiber membrane was conducted by using a thermal gravimetric analyzer (TG209F3, Germany) in an air atmosphere, and the heating rate was 10 °C min^−1^. Tensile properties were measured using a universal testing machine (AG–X plus, Shimadzu Instruments) at a loading rate of 1 mm min^−1^. The sheet resistance (*R*_s_) was examined using an RTS-8 four-point probe, and the corresponding electrical conductivity (*σ*) was calculated using the equation: *σ* = 1/(*d*·*Rs*) (*d* is the film thickness) [37]. The thermal conductivity was measured by a thermal constant analyzer (TPS2500S, Hot Disk AB, Sweden). The EMI shielding performance of the MXene@c-MWCNT_x:y_ and SMC_x_ were measured using an Agilent PNAN5244A vector network analyzer at room temperature in frequency ranges of 8.2–12.4 GHz (X-band), and the test samples were circular films with a diameter of 4 cm.

### EMI Shielding Testing

In this test, the center of the inside sides of the test fixture is a rectangular cavity with a length of 2 cm and a width of 1 cm. The samples were made into a circle with a diameter of 4 cm to ensure that the sample can completely cover the cavity and successfully complete the experiment. When the experiment was carried out, the fixture was forcefully clamped to avoid serious EM wave exposure, so as to obtain reliable experimental data. The power coefficients of reflection (*R*), absorption (*A*) and transmission (*T*), as well as the total EMI SE (SET), absorption SE (*SE*_A_), and reflection SE (*SE*_R_) were calculated as follows [38]:

1 $$R = \,|S_{11} \left| {\phantom{i}}^{2} ,T = \,\right|S_{21} |^{2}$$

2 $${1 } = A + R + T$$

3 $$SE_{{\text{T}}} = \, SE_{{\text{A}}} + \, SE_{{\text{R}}} + \, SE_{{\text{M}}}$$

4 $$SE_{{\text{R}}} = - 10 \, \log |1 - R|, \, SE_{{\text{A}}} = - 10 \, \log |T/(1 - R)|$$

where *S*_11_ represents forward reflection coefficient, *S*_12_ represents reverse transmission coefficient, and *SE*_M_ represents the multiple reflection SE between the two surfaces of the film [39]. When *SE*_T_ ⩾ 15 dB, the *SE*_M_ can be ignored [40].

### Thermal Insulation Testing

In addition to directly reflecting the thermal conductivity of the sample with the thermal constant analyzer, the thermal insulation performance of the sample was visually tested with the infrared thermal imager (E60, FLIR, America) under simulated high-temperature and cold environments. Here, infrared thermal imager is used to record real-time surface temperature changes of different samples over time. In order to ensure the reliability of the data, the initial temperature of the samples under similar experiment (high-temperature or cold environment) needs to be consistent.

For high-temperature environment testing, Xenon lamp devices (CEL-PE300L-3A, China Education Au-light Co., Ltd, China) that release simulated sunlight can provide continuous high-temperature environment. During the experiment, the circular sample with a diameter of 4 cm was placed 15 cm perpendicular to the 300 W Xenon lamp source (Fig. S2a).

For low-temperature environment testing, a cylindrical glass container (with an inner diameter of 15 cm and a depth of 9 cm) filled with ice cubes is freshly removed from the refrigerator and covered with a layer of plastic wrap to keep the sample from getting soggy (Fig. S2b).

## Results and Discussion

### Characterization

**Fig. 1:**
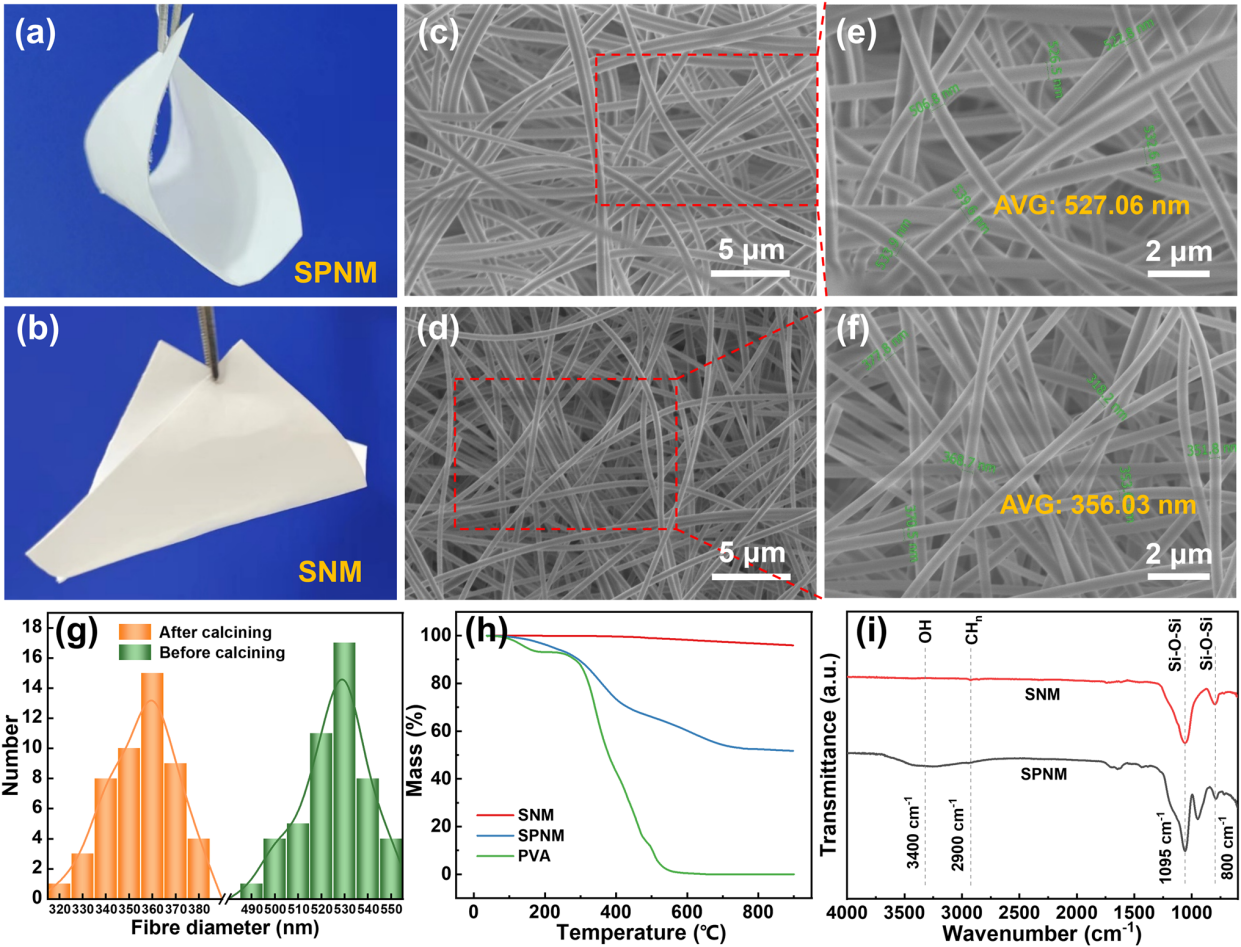
**a, b** Digital photographs, **c-f** SEM images and **g** fiber diameter size distribution of SPNM and SNM. **h** TG curves of SPNM, SNM and PVA. **i** ATR-FTIR spectra of SPNM and SNM

As shown in Fig. 1a, b, compared to the digital photographs of SPNM, the SNM obtained after calcination still maintain great flexibility and its surface becomes smooth and dense. Figure 1c, d shows the SEM test results of SPNM and SNM at the same magnification, respectively. It can be seen that the fiber distribution of both is relatively uniform and the fiber diameter of SNM has been significantly decreased. By further magnifying the scanning magnification, from Fig. 1e and f, it can be clearly seen that the fiber diameter was also uniform. Fifty fibers were selected from SPNM and SNM respectively for diameter statistical analysis, and it was found that the diameter distribution of both fibers followed a normal distribution (Fig. 1g). Specifically, the fiber diameter of SPNM is generally distributed in the range of 490–550 nm, and the fiber diameter of SNM is generally distributed in the range of 350–360 nm. After statistical calculation (Table S1), the average fiber diameter of SNM decreased from 527.06 nm in SPNM to 356.03 nm.

Figure 1h presents that the TGA curves of PVA, SPNM and SNM from 30 to 900 °C in the air atmosphere. The TGA curve of PVA shows that it can be completely decomposed before 600 °C, which is consistent with the conclusion of other studies [41, 42]. It can be seen from the TGA curve of SPNM that the weight loss of SPNM is mainly concentrated in two stages: the range of 100–400 °C with a mass loss of 27% and the range of 400–750 °C with a mass loss of 20%. The former is mainly attributed to the removal of water molecules and part of PVA, while the latter is mainly due to the removal of residual organic matter. In addition, the TGA curve of the SNM showed that its mass has hardly changed throughout the entire testing temperature range. Combined with the TGA of PVA and SPNM, it can be preliminarily concluded that the SNM obtained after heat treatment at 800 °C have a high-temperature resistance and no organic components.

So as to further analyze the components of SNM and SPNM, they were characterized by ATR-FTIR (Fig. 1i). In FTIR spectra of SPNM, the broad peaks at around 3400 and 2900 cm^−1^ were assigned to the − OH bonds and the CH_n_ groups, respectively [43–45]. However, the FTIR spectra of SNM do not have the above two characteristic peaks, indicating that organic matter (such as PVA, residual TEOS and H_2_C_2_O_4_) in SPNM is completely removed after being calcined at 800 °C. Moreover, the spectra only show obvious peaks near 1095 and 800 cm^−1^, which correspond to the tensile vibration of Si–O-Si bonds [46]. The above results comprehensively indicate that SNM are mainly composed of ceramic SiO_2_ phase. Combined with TGA results, it has been proven that after heat treatment at 800 °C, organic components in SPNM are completely removed, and the resulting SNM are only composed of inorganic components of SiO_2_. This conclusion also effectively explains the obvious decrease in fiber diameter from SPNM to SNM.

**Fig. 2:**
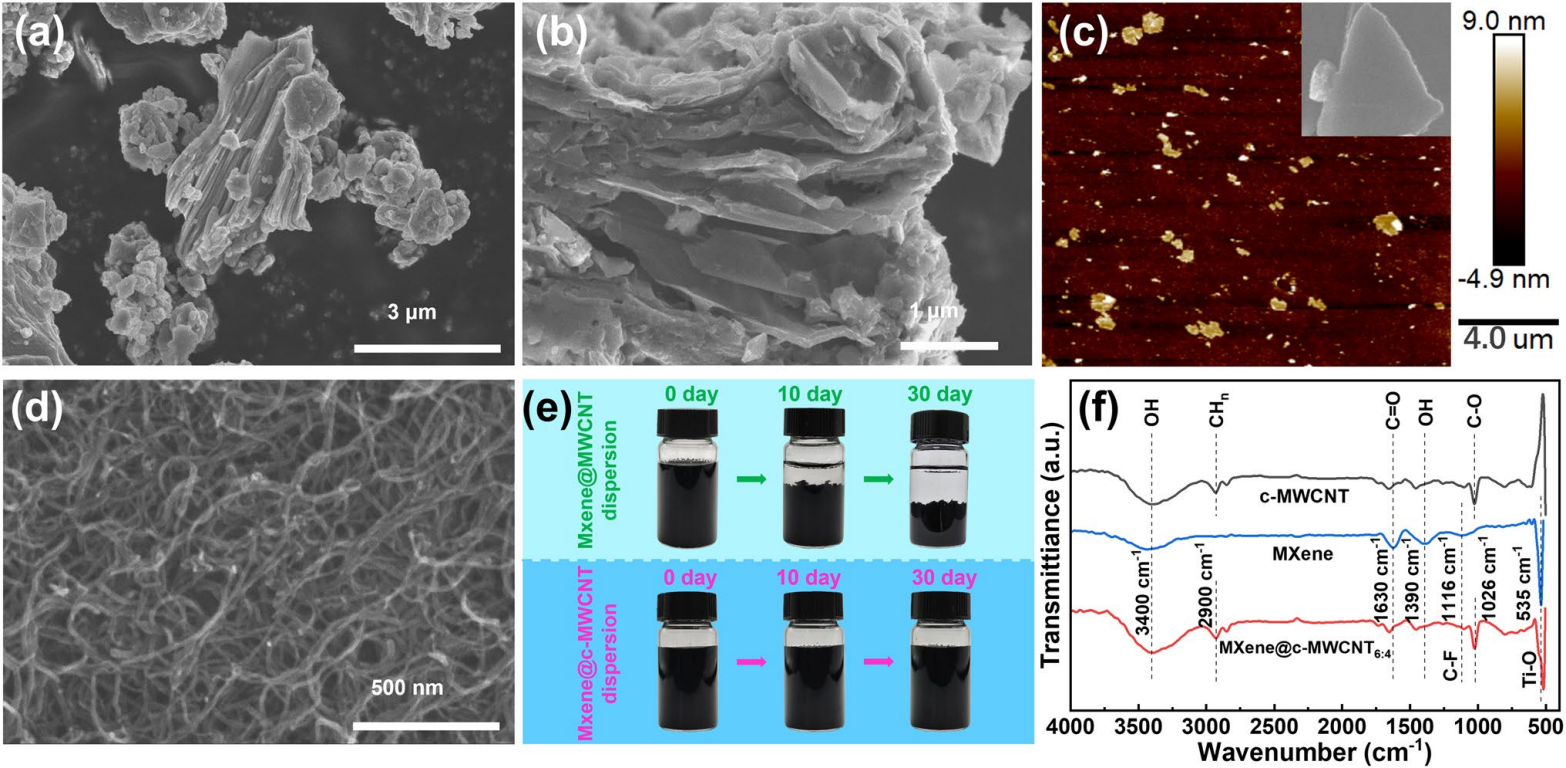
**a** SEM image of Ti_3_AlC_2_. **b** SEM image of multi-layer MXene without ultrasonication. **c** AFM image and SEM image (inset) of MXene monolayer. **d** SEM image of c-MWCNT. **e** Digital photograph of MXene@MWCNT_6:4_ and MXene@c-MWCNT_6:4_ dispersions for different static times. **f** ATR-FTIR spectra of MXene, c-MWCNT and MXene@c-MWCNT_6:4_

Ti_3_C_2_T_x_ MXene nanosheets with a high specific surface area are usually prepared by acid etching and ultrasonic exfoliation [47–49]. In this experiment, after the middle aluminum layer was etched off by HF, the compact massive carbon-aluminum-titanium (Ti_3_AlC_2_ MAX) particles (Fig. 2a) evolved into multilayered Ti_3_C_2_T_x_ MXene with an accordion structure (Fig. 2b). Further ultrasonic stripping resulted in ultra-thin monolayer Ti_3_C_2_T_x_ MXene (Fig. 2c) [50]. Figure 2d shows the SEM microscopic morphology of c-MWCNT, which are uniformly distributed and correspond to a product description with an inner diameter of 3–5 nm, an outer diameter of 8–15 nm, and a length of 5–15 μm. Figure 2e presents the standing experiments of MXene@MWCNT_6:4_ and MXene@c-MWCNT_6:4_ dispersions, respectively. It can be clearly seen that after being placed for 10 days, the former shows a distinct deposition separation phenomenon, while the latter still remains a uniform dispersion. After 30-day placement, more than half of the solid deposits appeared in the former, while the latter remained almost unchanged. In comparison, it can be preliminarily concluded that the active functional groups (-OH, -F, C = O, etc.) of MXene and c-MWCNT are well combined, thereby improving the overall dispersibility and antioxidant properties.

**Fig. 3:**
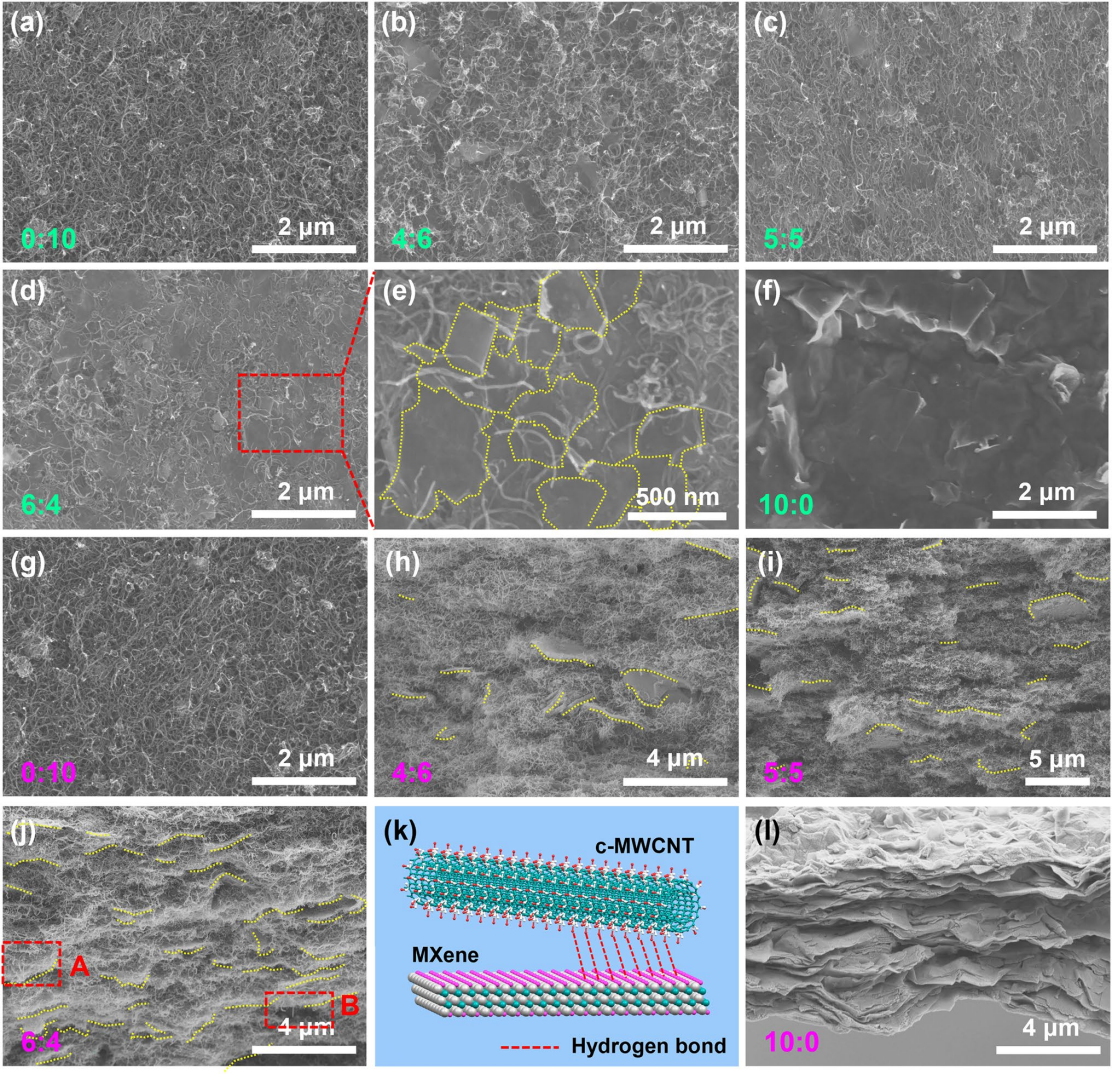
SEM images of **a-f** the surface and **g-j**, **l** the cross section of MXene@c-MWCNT_x:y_ with different weight ratio of MXene and c-MWCNT. **k** Diagram of hydrogen bond between MXene and c-MWCNT

The major valence bonds of MXene, c-MWCNT and MXene@c-MWCNT_x:y_ were further analyzed by ATR-FTIR. As shown in Fig. 2f, the FTIR spectrum of MXene shows typical representative vibrational peaks, which are, respectively, attributed to the − OH bonding near 3400 and 1390 cm^−1^, C = O bonding near 1630 cm^−1^, C − F bonding near 1116 cm^−1^, and Ti − O terminal group near 535 cm^−1^ [30, 51]. In addition to the aforementioned − OH and C = O characteristic peaks, the FTIR spectrum of c-MWCNT also contains C − O bonding near 1026 cm^−1^ and CH_n_ groups near 2900 cm^−1^ [52]. The FTIR spectrum of MXene@c-MWCNT_6:4_ contains all the characteristic peaks of MXene and c-MWCNT, indicating the formed effective valence bond binding between them. It is worth noting that the FTIR spectrum of MXene@c-MWCNT_6:4_ is similar to that of c-MWCNT (except for the Ti − O characteristic peak). This is because when MXene and c-MWCNT are mixed together, the latter wraps the former (Fig. 3j), resulting in most of the infrared beams to preferentially contact and reflect with c-MWCNT during testing.

In order to analyze the microstructures of MXene@c-MWCNT_x:y_ more thoroughly, SEM characterizations were carried out from both the surface and the cross section. As can be seen from the surface SEM images (Fig. 3a, b, c, d), with the increase in the relative content of MXene, the number of transparent MXene sheets distributed on the surface of MXene@c-MWCNT_x:y_ is gradually increased. Especially when the mass ratio of MXene:c-MWCNT reaches 6:4, the MXene sheets are in contact with each other (Fig. 3e), which helps to improve the overall electrical conductivity of MXene@c-MWCNT_6:4_.

For the SEM images of the cross sections, similarly, the number of MXene sheets embedded in c-MWCNT gradually increases with the increase in their content (Fig. 3g, h, j). From Fig. 3j, it can be seen that MXene sheets distributed at MXene@c-MWCNT_6:4_ are dense and uniform. In addition, region A indicates that MXene sheets are enveloped by a large amount of c-MWCNT, and region B reveals that the surrounding linked c-MWCNT will be taken away when MXene is pulled out. The above two phenomena imply a strong binding force between MXene and c-MWCNT, which is provided by the hydrogen bond formed between their active functional groups (Fig. 3k) [53]. Based on the above analysis, one-dimensional c-MWCNT and uniformly embedded two-dimensional MXene form a “hand in hand” three-dimensional wire junction structure, which provides a theoretical basis for MXene@c-MWCNT_6:4_ with good electrical conductivity and mechanical properties. From Fig. 3f, l, it can be seen that a pure MXene film is formed by stacking rigid and fluffy MXene sheets.

### Mechanical Tensile Performance

**Fig. 4:**
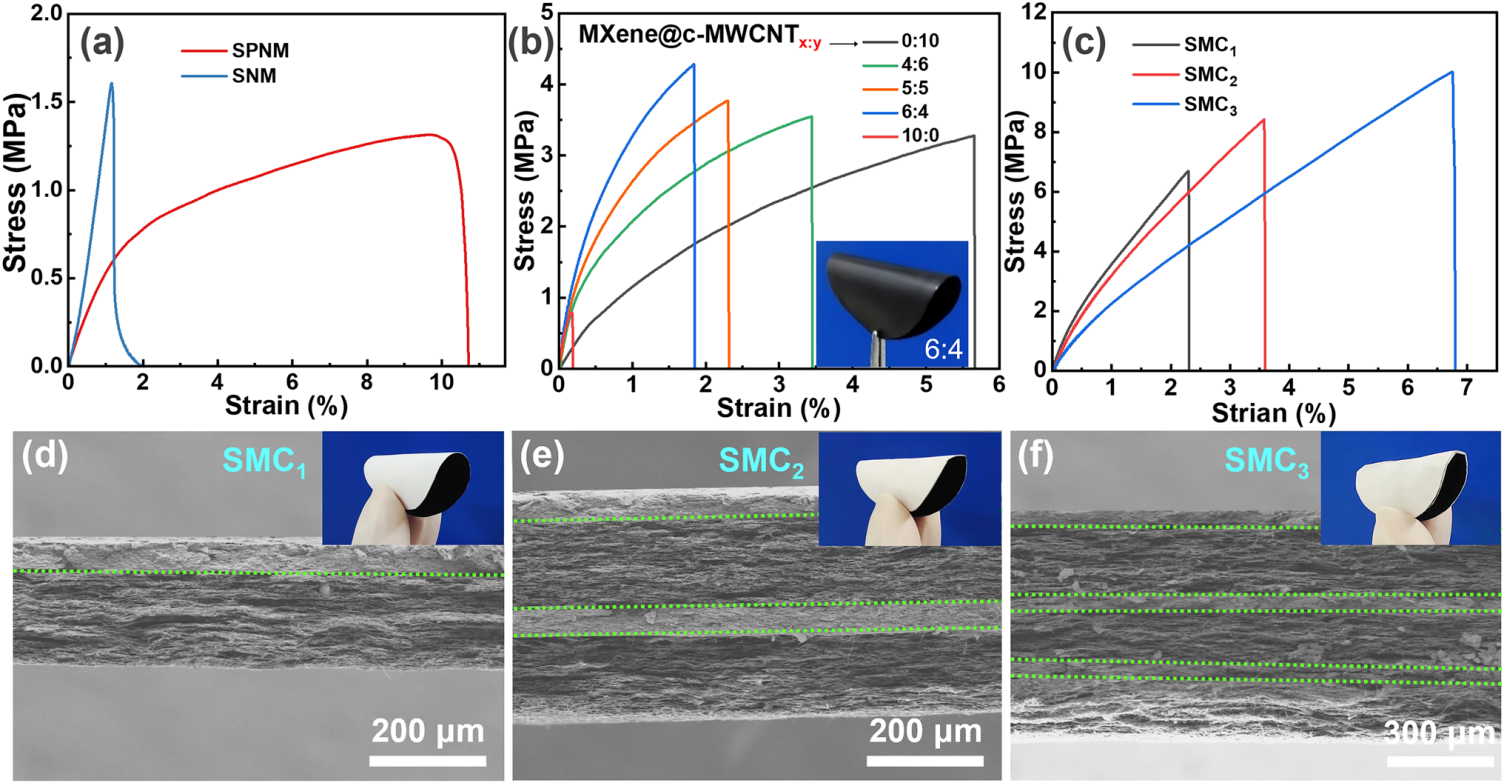
Typical stress–strain curves of **a** SPNM and SNM, **b** MXene@c-MWCNT_x:y_, and **c** SMC_x_. **d-f** SEM cross-sectional images of SMC_x_ and corresponding digital photographs

Both good mechanical tensile strength and flexibility are important indicators for the practical application of fiber-based composites [54–56]. As demonstrated in Fig. 4a, the tensile strength and modulus of SNM obtained by calcination are greater than that of SPNM. Figure 4b reveals that the tensile strength of MXene@c-MWCNT_x:y_ is significantly higher than that of pure MXene and c-MWCNT films, and the tensile strength of hybrid films increases with the increase in MXene content. When the weight ratio of MXene to c-MWCNT is 6:4, MXene@c-MWCNT_6:4_ reaches the maximum tensile strength of 4.28 MPa, which is mainly attributed to the strong interfacial adhesion between MXene nanosheets and c-MWCNT through hydrogen bonding and π-π interaction [57]. However, the maximum strain of the hybrid film decreases with the increase in MXene content, which is mainly due to the weak van der Waals forces between the rigid MXene nanosheets. Through the complementary effect of c-MWCNT, it can be seen from Fig. 4b (inset) that MXene@c-MWCNT_6:4_ still has a good flexibility.

From Fig. 4c, it can be clearly seen that the tensile strength of SMC_x_ composite film composed of SNM/MXene@c-MWCNT_6:4_ (unit layer) has been greatly improved, especially for SMC_3_ prepared by bonding three unit layers with 5 wt% PVA, which has a mechanical tensile strength of 10.01 MPa. Figure 4d, e, f shows the tensile cross sections of SMC_1_, SMC_2_, and SMC_3_, it can be seen that SNM and MXene@c-MWCNT_6:4_ are tightly coupled, as well as between different unit layers. As shown in the insertion, the SMC_3_ containing three unit structures still maintains good bendability. It is worth mentioning that the SMC_x_ obtained in this work realizes the micrometer level in the thickness direction (Table S2), which can greatly save space and enhance the value of practical application.

### Electromagnetic Interference Shielding Performance

**Fig. 5:**
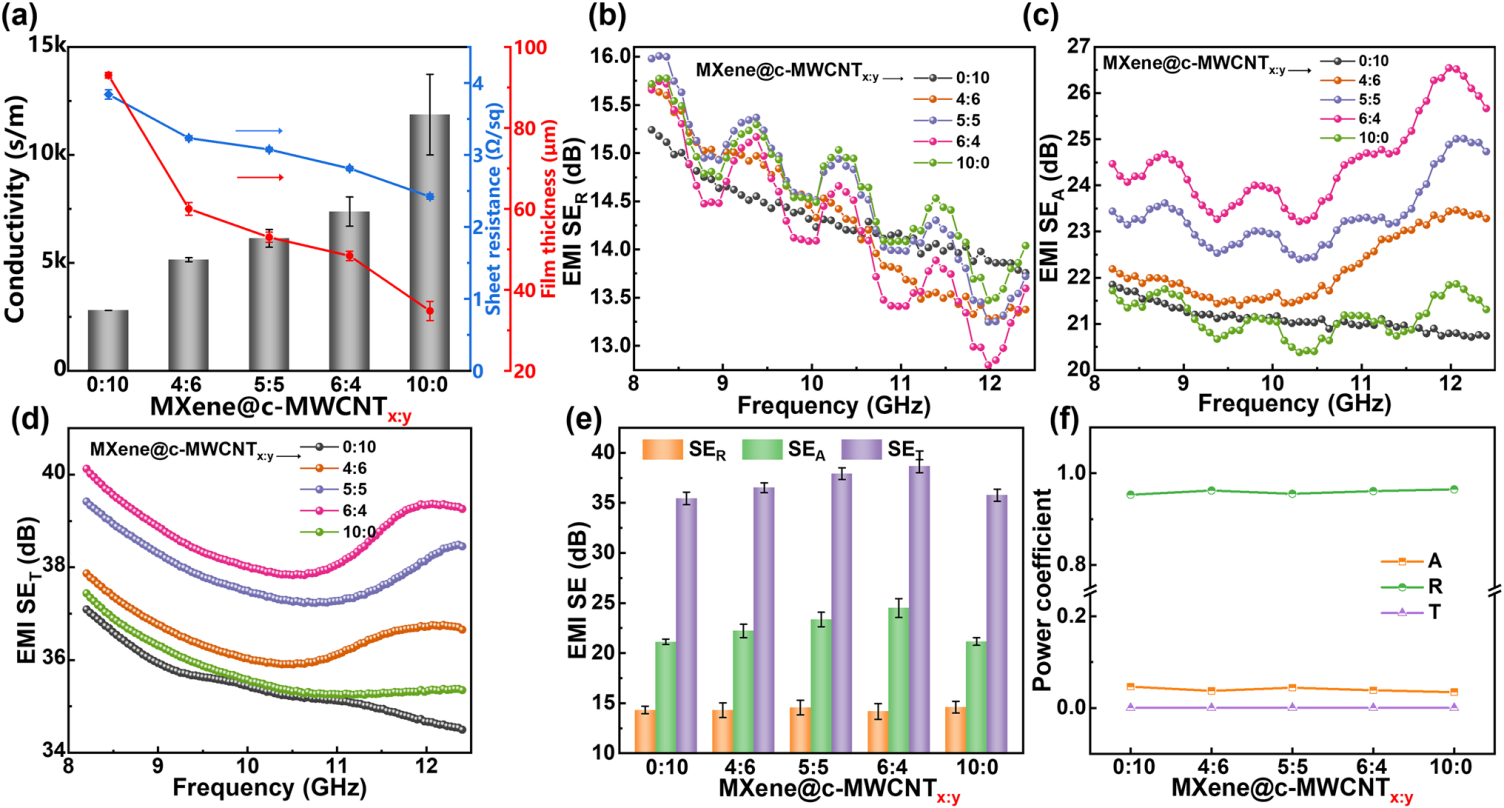
**a** Conductivity, sheet resistance and film thickness, **b-d** EMI *SE*_T_, *SE*_R_, *SE*_A_ performance in the X-band, **e** average EMI *SE*_T_, *SE*_A_, *SE*_R_ values and **f** power coefficients of MXene@c-MWCNT_x:y_

Electrical conductivity is one of the main factors affecting the performance of EMI shielding [58, 59]. MXene@c-MWCNT_x:y_ serve as the main contributor of EMI shielding in this work, as shown in Fig. 5a, with the increase in MXene content, the conductivity of MXene@c-MWCNT_x:y_ is significantly enhanced (MXene@c-MWCNT_6:4_ reaches 7378 S m^−1^), while the sheet resistance and thickness of hybrid film are obviously reduced (Fig. S3 and Table S3). Here, although the pure MXene film has the highest electrical conductivity (11,869 S m^−1^), its poor mechanical stretchability limits its further application.

In Fig. 5b, the SE reflection (*SE*_R_) curve of pure c-MWCNT film in the X-band is relatively stable, while the *SE*_R_ curves of the pure MXene film and hybrid films present wavy shape, and the EMI *SE*_R_ interval values of all films are approximately concentrated between 13–15 dB. Combining the formula *SE*_R_ (dB) = 20 log(*Z*_*0*_*/*4*Z*_*1*_), where *Z*_*0*_ and *Z*_*1*_ are the impedance of free space and shielding material, respectively [60], it can be concluded that under the premise of constant spatial impedance, the two different SE_R_ curve trends are attributed to the uniform diameter distribution of one-dimensional c-MWCNT and the non-uniform diameter of two-dimensional MXene, respectively.

As shown in Fig. 5c, the SE absorption (*SE*_A_) value of the hybrid film in the X-band is enhanced with the increase in MXene content and reaches the maximum value when the ratio of MXene to c-MWCNT is 6:4. The EMI *SE*_A_ is mainly determined by the shielding thickness (*d*) and skin depth (*δ*) [61], and their general relationship is *SE*_A_ (dB) = 20(*d/δ*)log*e* = 8.686(*d/δ*), where *δ* is skin depth and defined as the electromagnetic energy decreases to *e*^−1^ of the incident wave, it is described as *δ* = (*πfµσ*)^−1/2^ if *σ* >  > *2πfε*_*0*_, in which *σ* is the electrical conductivity, *ε*_*0*_ is the vacuum permittivity, and permeability *µ* = *µ*_*0*_*µ*_*r*_ (*µ*_*0*_ = 4π × 10^−7^ H m^−1^, *µ*_*r*_ = 1), so shielding thickness (*d*) and the electrical conductivity (*σ*) are the critical factors for *SE*_A_ [62]. According to the above analysis, it can be concluded that in the process of gradual change of MXene/c-MWCNT mass ratio from 0:10 to 6:4, the progress of *SE*_A_ mainly benefits from the increase in electrical conductivity (*σ*) (Fig. 5a), but when the ratio transitions from 6:4 to 10:0, the decrease in *SE*_A_ is mainly attributable to a sharp reduction in the thickness (*d*) of the shielding film (Table S3). In addition, as shown in Fig. 5d, the change trend of EMI shielding effectiveness total (*SE*_T_) is consistent with that of *SE*_A_.

The average values of *SE*_T_, *SE*_A_, and *SE*_R_ in the X-band of the sample can effectively disclose its electromagnetic shielding mechanism. It can be clearly seen from Fig. 5e that the *SE*_T_ values of MXene@c-MWCNT_x:y_ are all greater than 30 dB, which indicates that they can meet the actual requirements in various fields [63]. Combined with the double advantages of electrical conductivity and thickness, the *SE*_T_ average value of MXene@c-MWCNT_6:4_ is as high as 38.66 dB. According to the shielding efficiency formula *η* (%) = 100–100(1/10^*SE*/10^) [62], MXene@c-MWCNT_6:4_ can shield 99.98% of incident electromagnetic wave. In addition, the *SE*_R_ values of all films are stable in the range of 15 dB, indicating that more than 90% of the incident electromagnetic wave is shielded by reflection [64].

Although the *SE*_A_ values of the shielding films in Fig. 5e are higher than the *SE*_R_ values, most electromagnetic waves are reflected before entering the shielding layer, so the power coefficient of EMI shielding needs be further analyzed [65]. The power coefficient includes absorption coefficient (*A*), reflection coefficient (*R*) and transmission coefficient (*T*), which are used to evaluate the ability of EMI shielding materials to absorb, reflect and transmit electromagnetic waves, respectively [66]. Figure 5f shows that the T values of the shielding films are close to 0, indicating that MXene@c-MWCNT_x:y_ can shield almost all incident electromagnetic waves. At the same time, the much higher *R* values than *A* values indicate that MXene@c-MWCNT_x:y_ mainly follow a reflectance-based electromagnetic shielding mechanism.

**Fig. 6:**
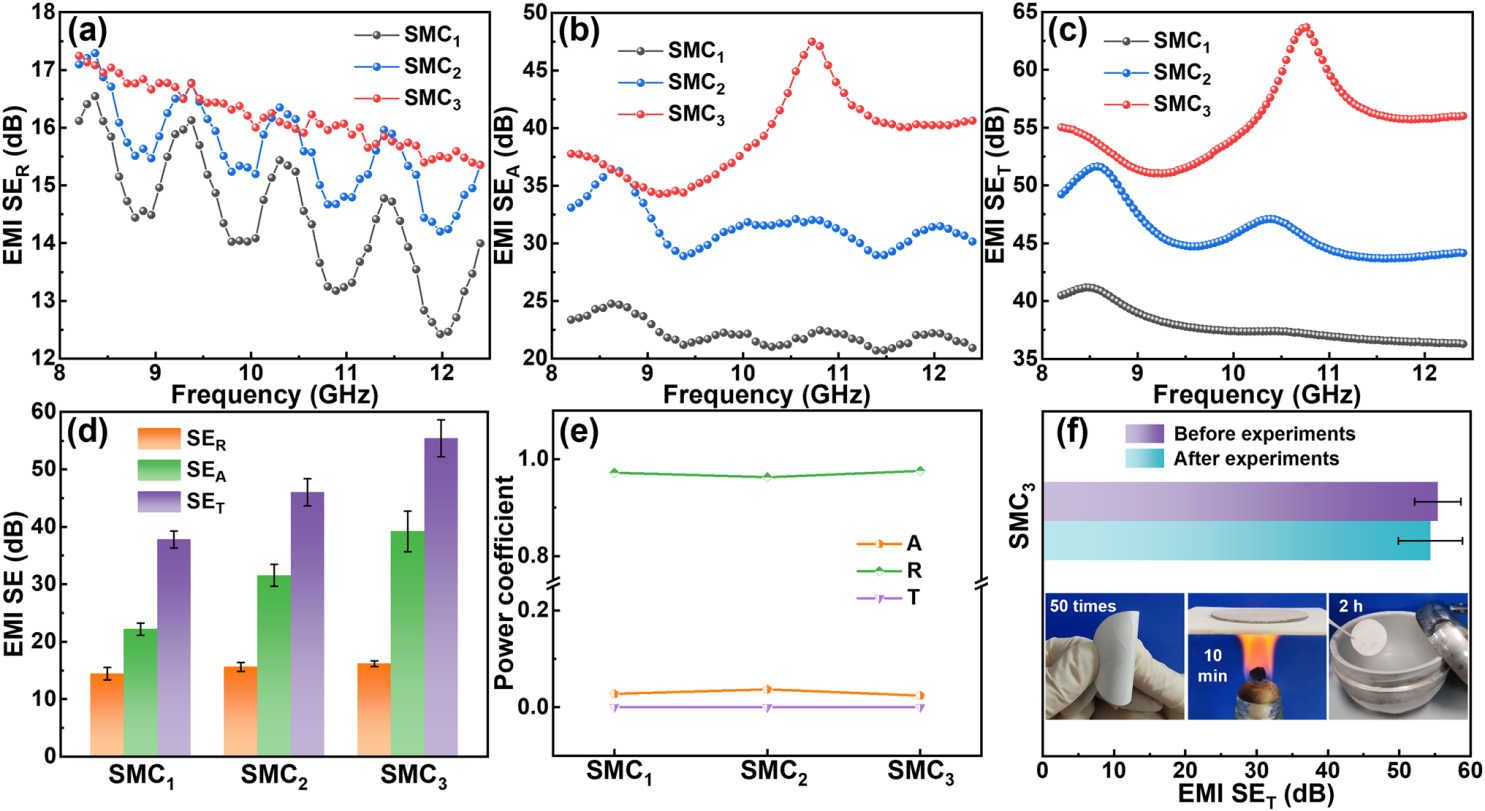
**a**-**c** EMI *SE*_R_, *SE*_A_, and *SE*_T_ performance, **d** average EMI *SE*_T_, *SE*_A_, *SE*_R_ values and **e** power coefficients of SMC_x_. **f** EMI shielding performance of SMC_3_ after a series of extreme environment tests

The number of structural unit layers contained in SMC_x_ is an important factor affecting the electromagnetic shielding performance. As shown in Fig. 6a, b, c, the *SE*_R_ and *SE*_A_ of composite film SMC_x_ in X-band both rise with the increase in the number of structural unit layers, and *SE*_T_ of SMC_3_ is greatly improved compared with SMC_1_ and SMC_2_. SMC_1_ can only reflect and absorb electromagnetic waves on one side, while the EMI shielding mechanism of SMC_2_ and SMC_3_ also includes multiple reflections and multiple absorption, so the *SE*_T_ increases with the increase in the number of layers of structural units. Therefore, the average *SE*_T_ of SMC_1_, SMC_2_, and SMC_3_ in Fig. 6d is 37.80, 46.00, and 55.40 dB, respectively. After calculation, SMC_3_ can shield 99.99% of incident electromagnetic wave. Similar to MXene@c-MWCNT_x:y_, it can be seen from Fig. 6e that the main shielding mechanism of SMC_x_ is also reflection. In order to explore the durability of the electromagnetic interference shielding characteristics of SMC_3_, a series of extreme environment tests have been carried out. As shown in Fig. 6f, after being bent 50 times, baked at high-temperature (about 500 °C) for 10 min, and frozen in liquid nitrogen for 2 h, the average *SE*_T_ value of SMC_3_ is still as high as 54.37 dB, indicating that the SMC_x_ has excellent EMI shielding durability.

### Thermal Insulation Performance

**Fig. 7:**
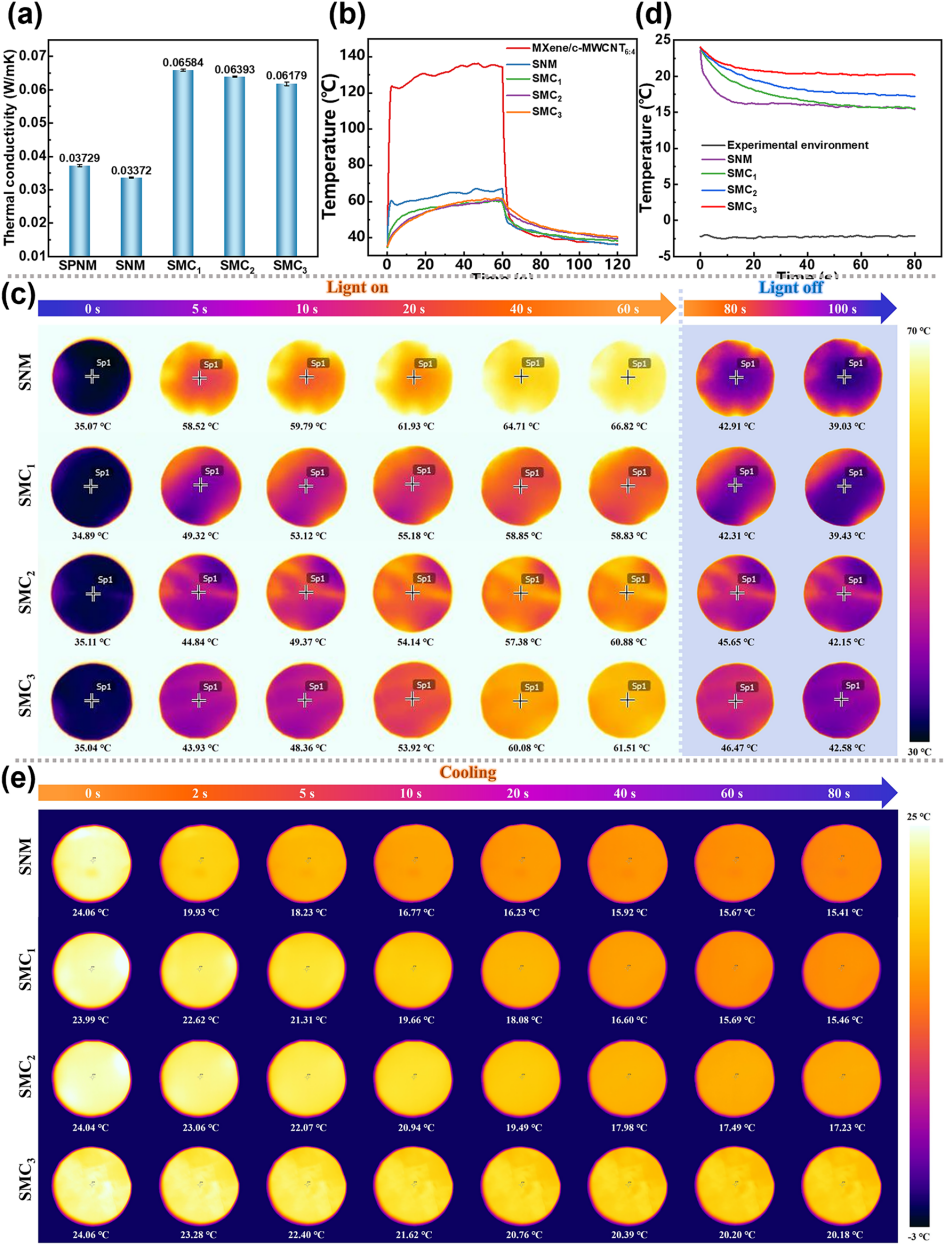
**a** Thermal conductivity of SPNM, SNM and SMC_x_. **b** Temperature–time curves and **c** corresponding infrared images of MXene@c-MWCNT_6:4_, SNM and SMC_x_ in high-temperature environment. **d** Temperature–time curves and **e** corresponding infrared images of SNM and SMC_x_ in extreme cold environment

For composite films (SMC_x_), MXene@c-MWCNT_6:4_ serves as a functional layer for EMI shielding (Fig. 5), while SNM layer gives an excellent thermal insulation performance. In Fig. 7a, the thermal conductivity of SNM (0.034 W m^−1^ k^−1^) is lower than that of SPNM (0.037 W m^−1^ k^−1^), which is mainly due to the elimination of PVA and other organics in SPNM after high-temperature calcination, realizing SNM composed of pure SiO_2_ ceramic fiber. Although MXene (472 W m^−1^ k^−1^) and CNT (700 W m^−1^ k^−1^) have considerable theoretical thermal conductivity [32, 67], the SMC_x_ obtained by combining the SNM layer still maintain the ideal thermal insulation effect, and the thermal conductivities of SMC_1_, SMC_2_ and SMC_3_ are 0.066, 0.064, and 0.062 W m^−1^ k^−1^, respectively. This not only shows that SMC_x_ can effectively overcome the influence of the high thermal conductivity of the shielding film (MXene@c-MWCNT_6:4_) and maintain the excellent thermal insulation property of SNM, but also reveals that the thermal conductivity of SMC_x_ decreases gradually with the increase in the number of structural unit layers.

In order to more intuitively reflect the thermal insulation performance of SMC_x_, real-time monitoring of their thermal management performance in simulated high and low temperature environments is carried out using an infrared thermal camera [68]. For the high-temperature environment created by Xenon lamp, as shown in Fig. 7b, the surface temperature of MXene@c-MWCNT_6:4_ reaches above 120 °C instantaneously and is stabilized at about 135 °C after 40 s. In contrast, SNM showed excellent thermal insulation performance, with a surface temperature maintained at around 65 °C after 60 s. In addition, the thermal insulation effect of SMC_x_ obtained by bonding the shielding layer MXene@c-MWCNT_6:4_ and SNM with PVA has been further improved. From the infrared thermal imaging in Fig. 7c, it can be clearly seen that the final temperatures of SMC_x_ are all around 60 °C. It is worth mentioning that the thermal insulation performance of SMC_x_ did not gradually increase with the increase in structural units, mainly due to the certain photothermal conversion ability of MXene and CNT [69]. The natural cooling experiment after 60 s further verified the low thermal diffusion coefficient of SMC_x_.

For the low temperature environment created by ice, the temperature is maintained at about -2.5 °C. As shown in Fig. 7d, the cooling rates of SNM, SMC_1_, SMC_2_, and SMC_3_ decreased successively for different fiber films placed in the cold environment at the same time. Importantly, the temperature protection capacity of SMC_x_ at low temperature increased with the increase in the number of unit layers, corresponding to SMC_1_: 15.46 °C, SMC_2_: 17.23 °C and SMC_3_: 20.18 °C, respectively (Fig. 7e). Interestingly, the minimum temperature of SMC_1_ is similar to that of SNM, which should be because SMC_1_ only contains one layer of SNM, making its thermal insulation performance very close to that of SNM. In summary, SMC_x_ shows excellent thermal insulation and heat preservation for extreme thermal and cold environments, respectively.

### Dual-Effect Mechanism of EMI Shielding and Heat Insulation

**Fig. 8:**
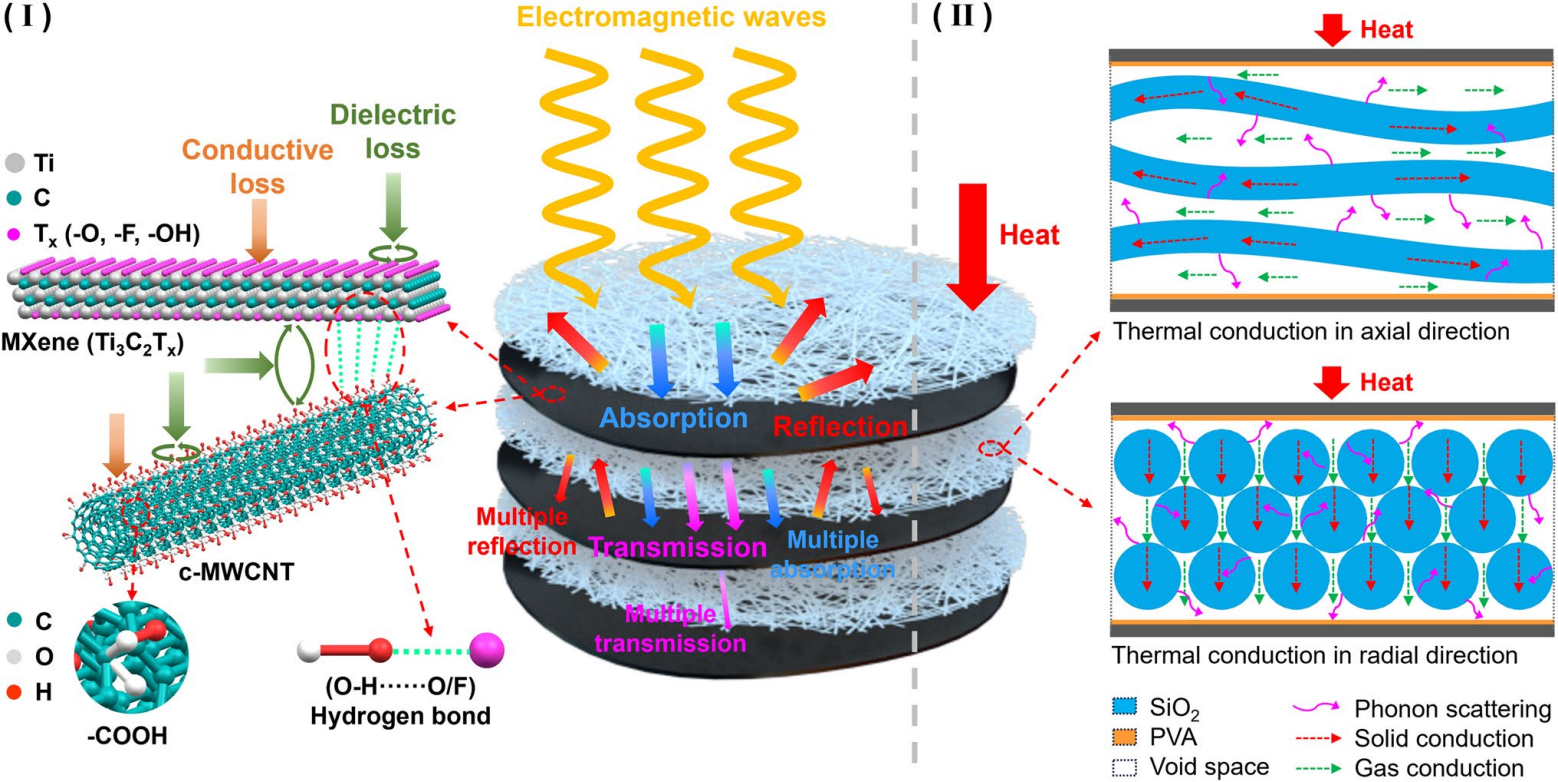
**I** EMI shielding and **II** thermal insulation mechanism diagram of SMC_x_

Combined with the above analysis results, SMC_x_ shows dual effects of EMI shielding and thermal insulation. For EMI shielding, MXene@c-MWCNT_6:4_ layer play a major role, as shown in Fig. 8I, when electromagnetic waves reach the surface of the shielding film, more than 90% of the electromagnetic waves are immediately reflected due to the impedance mismatching of the surface free electrons [70]. Next, the interaction between the incident electromagnetic waves and the high density of electrons and holes in the conductive layer causes conductive loss, weakening the power of the incident electromagnetic waves. In addition, the abundant active groups and heterogeneous interfaces provided by MXene and c-MWCNT induce both dipole and interfacial polarizations, further absorbing the energy of incident electromagnetic waves [71]. Therefore, after a series of reflections and absorption, only a very small amount of transmitted electromagnetic waves enter the next shielding layer. As a result, after multiple reflections and absorptions, almost no electromagnetic waves can penetrate the SMC_3_.

For thermal insulation, SNM layer play a major role, as shown in Fig. 8II, due to the small aperture between SiO_2_ fibers, the convective heat transfer can be almost negligible, so the total thermal conductivity (λ_t_) for SMC_x_ can be represented as the sum of contributions from three parts (λ_t_ = λ_s_ + λ_g_ + λ_r_) [72], i.e., the solid thermal conductivity (λ_s_), the gas thermal conductivity (λ_g_) and the radiation thermal conductivity (λ_r_). The thermal insulation mechanisms could be explained from the following four aspects: First, the extremely high length-to-diameter ratio and irregular winding of SiO_2_ fibers prolong the path of heat conduction in solids; moreover, the intrinsic thermal conductivity of SiO_2_ is low, leading a small λ_s_. Second, the high porosity of SNM makes the air heat transfer discontinuous in the whole space of SMC_x_, contributing a low λ_g_; third, the porous structure makes the infrared radiation multiple reflect and absorb, resulting in lowering of λ_r_; finally and most importantly, the phase interface between different solids as well as between solids and voids enhances phonon scattering [73].

## Conclusion

In this work, SNM with low thermal conductivity (0.034 W m^−1^ k^−1^) was prepared by electrospinning followed with calcination and played a decisive role in the thermal insulation performance of SMC_x_. The hybrid film composed of MXene and c-MWCNT was successfully prepared through vacuum assisted filtration, and the obtained MXene@c-MWCNT_x:y_ showed good EMI shielding performance. When the weight ratio of MXene to c-MWCNT was 6:4, MXene@c-MWCNT_6:4_ exhibited excellent comprehensive performance in terms of tensile strength (4.28 MPa) and EMI shielding (*SE*_T_ 38.66 dB). Finally, SMC_x_ with good EMI shielding and thermal insulation performance was successfully prepared by using 5 wt% PVA as an adhesive. Specifically, the thermal conductivities of SMC_1_, SMC_2_ and SMC_3_ are 0.066, 0.064, and 0.062 W m^−1^ k^−1^, and their EMI SE_T_ are 37.80, 46.00, and 55.40 dB respectively. In addition, the overall performance of SMC_x_ was improved with the increase in the number of structural unit layers. Importantly, SMC_x_ presents good durability in extreme hot and cold environments. The design ideas of this work have an important reference value for the development of special equipment such as manned space suits.

## Supplementary Information

Below is the link to the electronic supplementary material.

Supplementary file1 (DOCX 2351 kb)
