# Supplementary material for: MXene@c-MWCNT Adhesive Silica Nanofiber Membranes Enhancing Electromagnetic Interference Shielding and Thermal Insulation Performance in Extreme Environments
Source: Nanomicro Lett. 2024 May 14;16:195. doi: 10.1007/s40820-024-01398-1 (PMC11557810; doi:10.1007/s40820-024-01398-1)
Supplement: Supplementary file 1 — Supplementary file1 (DOCX 2351 kb) [file 40820_2024_1398_MOESM1_ESM.docx]

Supporting Information for

**MXene@c-MWCNT Adhesive Silica Nanofiber Membranes Enhancing Electromagnetic Interference Shielding and Thermal Insulation Performance in Extreme Environments**

Ziyuan Han^1^, Yutao Niu^2, 3^, Xuetao Shi^4^, Duo Pan^1,^ *, Hu Liu^1,^ *, Hua Qiu^4^, Weihua Chen^1, 5^, Ben Bin Xu^6^, Zeinhom M. El-Bahy ^7^, Hua Hou^6^, Eman Ramadan Elsharkawy^8^, Mohammed A. Amin^9^, Chuntai Liu^1^ and Zhanhu Guo^6,^ *

^1^ Key Laboratory of Materials Processing and Mold (Zhengzhou University), Ministry of Education; National Engineering Research Center for Advanced Polymer Processing Technology, Zhengzhou University, Zhengzhou 450002, P. R. China

^2^ School of Nano-Tech and Nano-Bionics, University of Science and Technology of China, Hefei 230026, P. R. China

^3^ Key Laboratory of Multifunctional Nanomaterials and Smart Systems, Advanced Materials Division, Suzhou Institute of Nano-Tech and Nano-Bionics, Chinese Academy of Sciences, Suzhou 215123, P. R. China

^4^ Shaanxi Key Laboratory of Macromolecular Science and Technology, School of Chemistry and Chemical Engineering, Northwestern Polytechnical University, Xi’an 710072, P. R. China

^5^ College of Chemistry & Green Catalysis Center, Zhengzhou University, Zhengzhou 450001, P. R. China

^6^ Mechanical and Construction Engineering, Faculty of Engineering and Environment, Northumbria University, Newcastle Upon Tyne, NE1 8ST, UK

^7^ Department of Chemistry, Faculty of Science, Al-Azhar University, Nasr City 11884, Cairo, Egypt

^8^ Department of Chemistry, Faculty of Science, Northern Border University, Arar, Saudi Arabia

^9^ Department of Chemistry, College of Science, Taif University, P.O. Box 11099, Taif 21944, Saudi Arabia

*Corresponding authors. E-mail: [panduonerc@zzu.edu.cn](mailto:panduonerc@zzu.edu.cn) (Duo Pan); [liuhu@zzu.edu.cn](mailto:liuhu@zzu.edu.cn) (Hu Liu); [zhanhu.guo@northumbria.ac.uk](mailto:zhanhu.guo@northumbria.ac.uk) (Zhanhu Guo)

**Supplementary Figures and Tables**


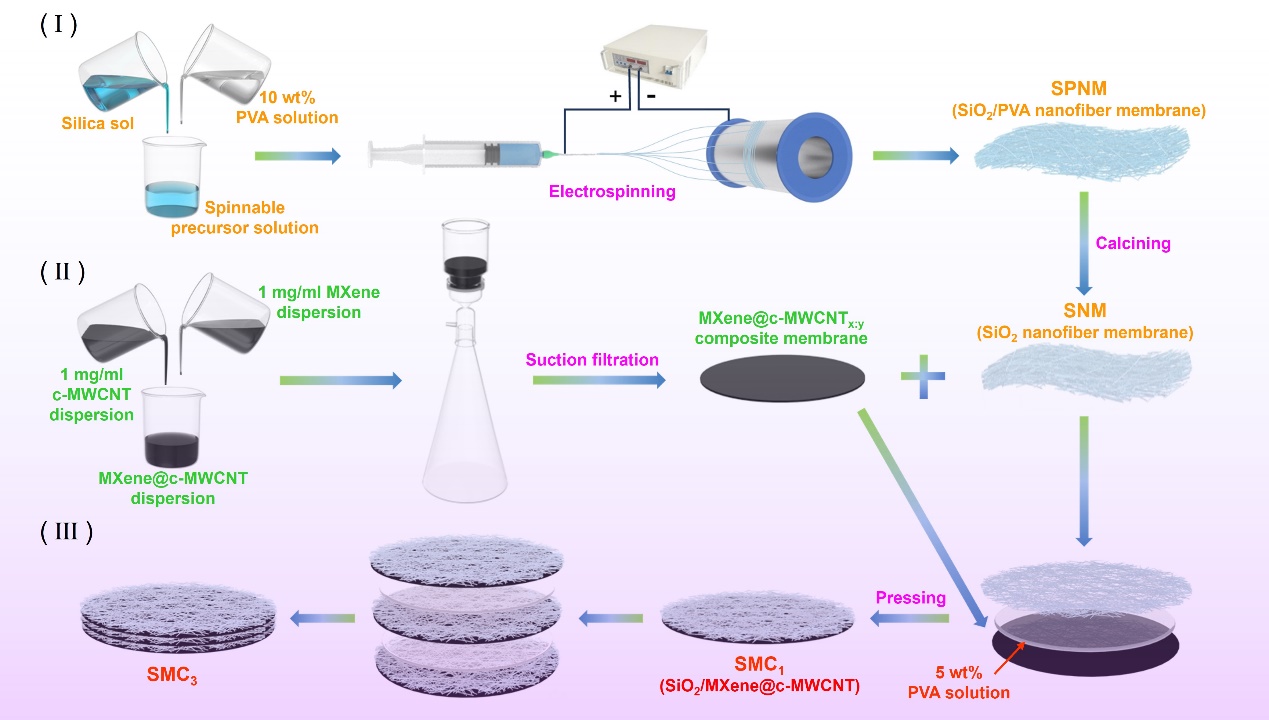


**Fig. S1** Schematic diagram of preparation of SMC_x_


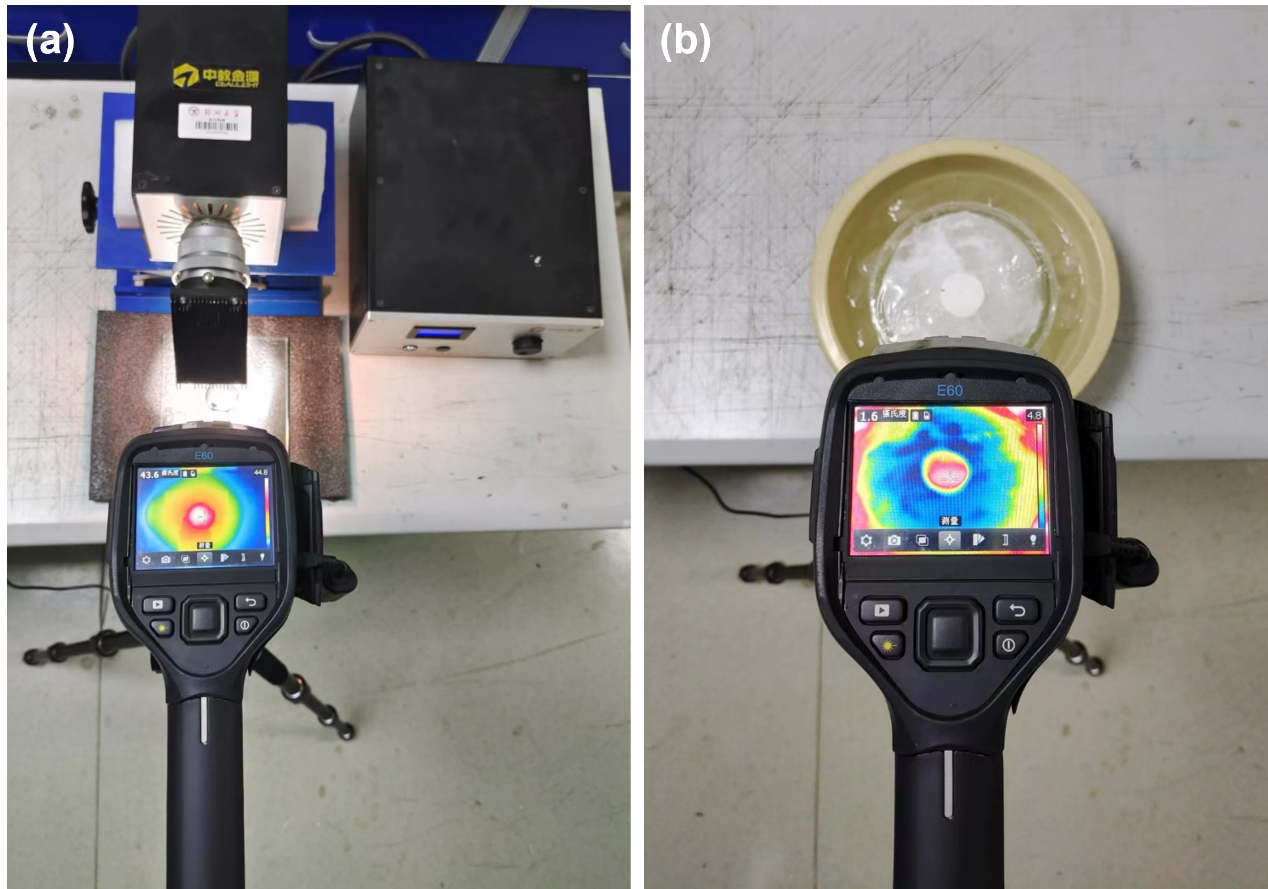


**Fig. S2** Thermal insulation testings in **a** high-temperature environment and **b** low-temperature environment, respectively


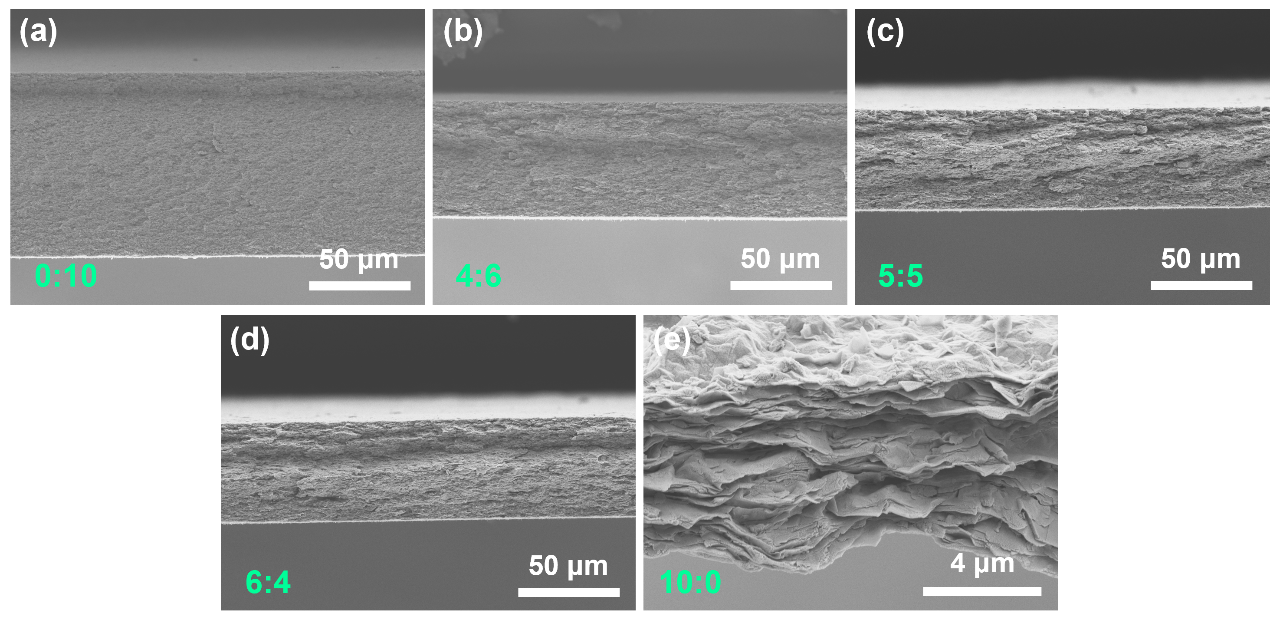


**Fig. S3 a-e** SEM images of the cross section of MXene@c-MWCNT_x:y_ with different weight ratio of MXene and c-MWCNT

| **Table S1a** Fiber diameter (nm) before calcination | |  |
| --- | --- | --- |
| Fiber diameter (nm) | AVG | |
| 494.7 498.2 501.7 502.9 504.6  505.9 510.8 511.3 512.2 514.9 516.5 518.4 519.1 520.6 521.1 521.8 522.5 523.1 523.5 524.3 524.7 525.9 526.4 527.2 529.2 529.2 529.8 531.0 531.6 531.7 532.6 532.6 532.9 533.4 533.7 534.1 534.4 534.8 535.7 536.0 539.8 540.7 542.2 543.0 544.2 544.3 547.8 549.1 552.3 554.6 | 527.06 | |
| **Table S1b** Fiber diameter (nm) after calcination | | |
| Fiber diameter (nm) | AVG | |
| 315.9 325.2 331.7 334.6 337.1 337.8 339.4 341.0 342.9 343.2 343.7 344.5 345.6 345.9 346.1 347.2 350.8 351.0 352.3 353.9 354.6 354.7 356.1 358.2 358.8 358.9 360.7 361.3 362.0 362.1 362.9 363.4 363.7 363.9 364.2 364.5 364.5 365.1 365.2 367.6 369.3 369.4 370.5 371.6 371.8 374.9 375.0 375.7 377.1 383.8 | 356.03 | |

| **Table S2** The average thickness (μm) of SMC_x_ and its components | | | | | |
| --- | --- | --- | --- | --- | --- |
| Samples | SNM | MXene@c-MWCNT_6:4_ | SMC_1_ | SMC_2_ | SMC_3_ |
| Average thickness | 169.7 | 50 | 224.4 | 446.8 | 672.3 |

| **Table S3** The average thickness (nm) of MXene@c-MWCNT_x:y_ | | | | | |
| --- | --- | --- | --- | --- | --- |
| X:Y | 0:10 | 4:6 | 5:5 | 6:4 | 10:0 |
| Average thickness | 93 | 60 | 55 | 50 | 34 |
